# Validating the Safe and Effective Use of a Neurorehabilitation System (InTandem) to Improve Walking in the Chronic Stroke Population: Usability Study

**DOI:** 10.2196/50438

**Published:** 2023-11-20

**Authors:** Kirsten Elisabeth Smayda, Sarah Hodsdon Cooper, Katie Leyden, Jackie Ulaszek, Nicole Ferko, Annamaria Dobrin

**Affiliations:** 1 MedRhythms Portland, ME United States; 2 Bold Insight Downers Grove, IL United States; 3 Eversana Burlington, ON Canada

**Keywords:** chronic stroke, walking, InTandem, MR-001, neurorehabilitation, human factors engineering, usability, rhythmic auditory stimulation, validation, neurotherapeutic

## Abstract

**Background:**

Persistent walking impairment following a stroke is common. Although rehabilitative interventions exist, few exist for use at home in the chronic phase of stroke recovery. InTandem (MedRhythms, Inc) is a neurorehabilitation system intended to improve walking and community ambulation in adults with chronic stroke walking impairment.

**Objective:**

Using design best practices and human factors engineering principles, the research presented here was conducted to validate the safe and effective use of InTandem.

**Methods:**

In total, 15 participants in the chronic phase of stroke recovery (≥6 months after stroke) participated in this validation study. Participants were scored on 8 simulated use tasks, 4 knowledge assessments, and 7 comprehension assessments in a simulated home environment. The number and types of use errors, close calls, and operational difficulties were evaluated. Analyses of task performances, participant behaviors, and follow-up interviews were conducted to determine the root cause of use errors and difficulties.

**Results:**

During this validation study, 93% (14/15) of participants were able to successfully complete the critical tasks associated with the simulated use of the InTandem system. Following simulated use task assessments, participants’ knowledge and comprehension of the instructions for use and key safety information were evaluated. Overall, participants were able to find and correctly interpret information in the materials in order to answer the knowledge assessment questions. During the comprehension assessment, participants understood warning statements associated with critical tasks presented in the instructions for use. Across the entire study, 3 “use errors” and 1 “success with difficulty” were recorded. No adverse events, including slips, trips, or falls, occurred in this study.

**Conclusions:**

In this validation study, people in the chronic phase of stroke recovery were able to safely and effectively use InTandem in the intended use environment. This validation study contributes to the overall understanding of residual use–related risks of InTandem in consideration of the established benefits.

## Introduction

Stroke is a major cause of disability and the second leading cause of death worldwide, with its incidence and prevalence expected to increase due to an aging population [1,2]. Many people in the chronic phase of stroke recovery (commonly defined as ≥6 months after stroke) experience walking impairment [3] and consider the ability to walk in their community as “either essential or very important” [4]. Walking rehabilitation can positively impact the well-being of stroke survivors and their families. It can also restore independence—a prospective study in the chronic stroke population reported that better walking ability was positively correlated with quality of life and the ability to live independently [5]. Clinical practice guidelines recommend various interventions for walking impairment, including physical therapy and braces such as an ankle foot orthosis [6-8]. Rhythmic auditory stimulation (RAS) is another clinically effective intervention for the rehabilitation of movements that are naturally rhythmic (eg, walking) [9]. RAS draws on a naturally occurring phenomenon called auditory-motor entrainment. During entrainment, an external auditory rhythm enables subconscious synchronization between the auditory and motor systems to drive coordinated movement patterns [10,11]. RAS has shown clinical benefits related to walking for patients with stroke across the subacute and chronic phases in many studies, several of which are randomized controlled trials (RCTs) [12-18]. In particular, speed, step length, cadence, balance, and dynamic postural stability [12,19,20] have been shown to improve in people who have had a stroke and receive RAS. In addition, the US Department of Veterans Affairs incorporated rhythmic auditory cueing into its clinical practice guidelines for the management of stroke rehabilitation in 2019 [21].

Currently, clinicians administer the RAS protocol in rehabilitation hospitals or clinics, while accessible at-home RAS-based interventions are nonexistent. Rehabilitation at home can carry benefits including half the risk of readmission, lower caregiver strain [22], reduced cost, and greater patient satisfaction [23] relative to hospital rehabilitation. For those in the chronic phase of stroke recovery, physical therapy and to a greater extent RAS can be difficult to access due to limited insurance coverage and the limited number of neurologic music therapists who deliver RAS. The lack of a solution for at-home walking rehabilitation is a critical gap in chronic stroke recovery, and it is imperative that solutions that are safe and effective to use are developed and delivered to address this unmet need.

To help close this gap, MedRhythms has designed MR-001 (InTandem, MedRhythms, Inc), a neurorehabilitation system that delivers a RAS-based intervention for chronic stroke walking impairment and is intended to be used independently at home.

## Methods

### System

**Figure 1:**
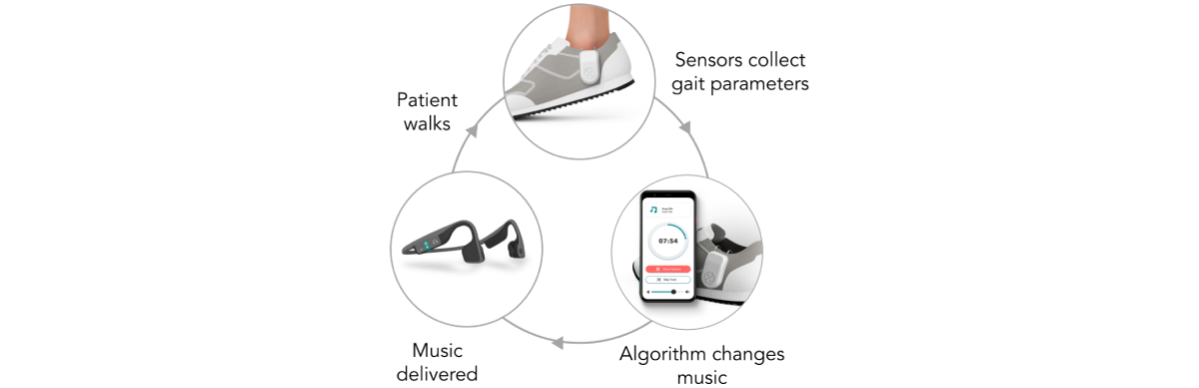
InTandem neurorehabilitation system.

The InTandem system (Figure 1) consists of 2 shoe-worn sensors that measure gait parameters, a locked touchscreen device preloaded with proprietary software, a headset, and charging equipment (not shown). The rhythmic stimulation used in InTandem is music. The music supplied has been screened for therapeutic benefit by a proprietary process that ensured the music met requirements for beat prominence and tempo. In developing InTandem, a patient-centered usability engineering process was followed to optimize for safe and effective use of InTandem according to the US Food and Drug Administration (FDA) guidance on human factors and usability engineering for medical device development [24]. Throughout this paper, InTandem might be referred to as “StridePlus” or “MR-001”—these were the nonproprietary names used during the development of the product. The product will be branded and commercialized as InTandem.

### Background on Formative Testing

**Figure 2:**
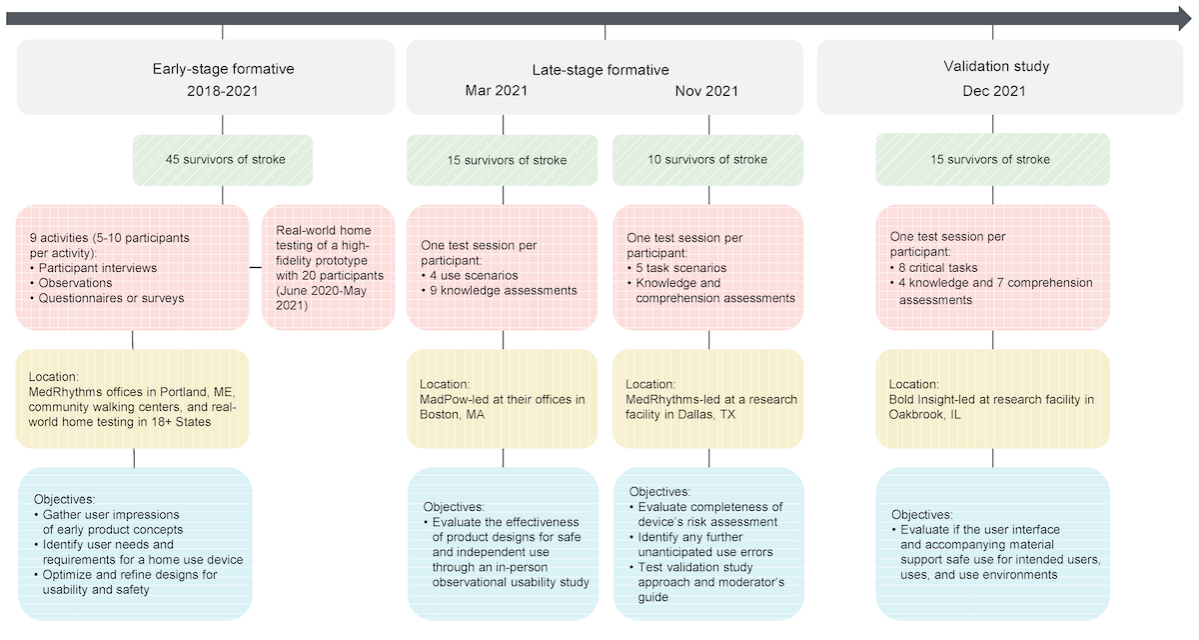
Overview of MR-001 usability testing across formative research activities and validation study.

This paper describes the methods and results of the human factors validation testing, which aimed to validate InTandem for safe and effective use. The testing was preceded by formative research activities across 2 phases to better understand stroke survivors’ needs for a home-use device, identify potential risks to patient safety, and iterate on system designs. A total of 70 unique stroke survivors with varied demographic backgrounds from both urban and rural locations across the United States were involved in the 2 formative research phases (see Figure 2 for more details about formative research activities). Some examples of learnings and iterations that occurred during the formative phases include (1) the need for improved sensor clips that allowed easier placement on sneakers that required less fine motor skill and hand strength and (2) the identification of which interactions with the product users needed the most education on and were less immediately intuitive out of the box Examples of results from the late formative phase included the need for improved instructions to walk and experience during the baseline walking part of a session, in which the sensors calibrate and measure cadence. A need for an improved user interface of the “pause” and “skip” buttons was also apparent in the late formative phase.

The validation study was conducted in December 2021 by an independent user experience and human factors research firm (Bold Insight). Bold Insight was selected to provide an unbiased third-party evaluation of the system’s usability and safety. This study assessed participant performance on all critical safety tasks to validate the usability of InTandem in the intended use population and environment (at home) and demonstrate that the product is not vulnerable to use errors that could lead to serious harm. Here, “critical tasks” are defined as “those that could cause serious harm in the event of a use error scenario or tasks with a severity of ‘moderate’ or greater identified in the use failure mode and effects analysis” (uFMEA; Multimedia Appendix 1). For the purposes of this paper, and in line with ISO 14971:2019 standard [24], “safety” is defined as the “freedom from unacceptable risk,” whereas “risk” is defined as the “combination of the probability of occurrence of harm and the severity of that harm.” In addition, according to IEC 62366 [25], “effectiveness” is defined as the “accuracy and completeness with which users achieve specified goals.” The study design followed a human factors validation protocol, and the study methods implemented were based on industry standards and FDA guidance [25-28]. Multimedia Appendix 1 provides definitions of terminology relevant to the study along with examples from the study [26,29].

### Ethical Considerations

The study protocol was reviewed and approved by the Biomedical Research Alliance of New York, an institutional review board (IRB #A20-02-508). Participants were reimbursed for out-of-pocket expenses incurred by being in the study.

### Participants, Setting, and System Set-Up

A total of 15 participants, with a mean age of 70 (SD 5.4; range 60-82) years and diagnosed as having had a stroke at least 6 months prior were recruited to meet the intended user population. Bold Insight managed screening and enrollment based on recruiting requirements. A broad group of patients with chronic stroke were recruited to ensure that the results reflected the differences in background and experiences of potential end users. Of note, although all participants reported not needing a caregiver to perform the tasks during screening, at the beginning of the session, 1 participant indicated their caregiver may help them in the real world, so that caregiver was invited into the session. A summary of enrolled patient characteristics can be found in Table 1.

**Table 1:** Summary of participant characteristics^a^.

Variable		Values (N=15)
Age (years), mean (SD)		70 (5.4)
**Gender, n (%)**		
	Male	9 (60)
	Female	6 (40)
**Education level, n (%)**		
	General educational development or high school	6 (40)
	PhD or higher	1 (7)
	Bachelor’s degree	5 (33)
	Trade school	2 (13)
	Master’s degree	1 (7)
**Time since the last stroke, n (%)**		
	6 months to <2 years	1 (7)
	2 years to <5 years	4 (27)
	5 years to <10 years	1 (7)
	10 years to <15 years	4 (27)
	15 years to <20 years	5 (33)
**Comfort level with technology, n (%)**		
	Basic tasks	3 (20)
	Okay	9 (60)
	Very comfortable	3 (20)
Caregiver needed, n (%)		0 (0)
Walking impairment, n (%)		13 (87)
Interested in improving the ability to walk, n (%)		14 (93)
Assisted walking devices, n (%)		2 (13)
Neurologic injury other than stroke, n (%)		0 (0)
Lower limb prosthetic, n (%)		0 (0)
Hearing impairment, n (%)		0 (0)
Severe aphasia, n (%)		0 (0)
Speech disorder, n (%)		0 (0)
Able to safely participate in a 5- to 7-minute walk, n (%)		15 (100)

^a^For the demographic variables that have 0%, this was intentional in order to replicate exclusion criteria used for clinical trials of InTandem.

To evaluate MR-001 use in the intended home environment, test sessions were performed at a usability laboratory that simulated a home setting and included items typically found in a personal living space (eg, table, chairs, and indoor ambient lighting). The 2-room research suite was equipped with a 1-way mirror, enabling researchers to observe the testing unobtrusively. The walking portion of the test sessions took place in a hallway outside the testing room that was kept private using screens.

### Procedures

**Figure 3:**
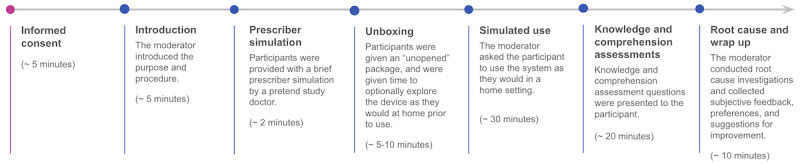
Session flow.

Each study session lasted approximately 90 minutes (see Figure 3 for session flow), and participants were scored on the completion of critical tasks that were assessed via 3 evaluation methods such as simulated use of the product, knowledge assessments, and comprehension assessments. Knowledge and comprehension assessment questions were used when tasks could not be simulated such as to verify whether the participants understood where they should use the device. Other noncritical tasks were completed as part of the procedure but were not scored. After consent was obtained and an introduction to the session was provided, each participant was provided with a prescribing scenario to simulate the experience of receiving a prescription of InTandem from a qualified health care provider. Specifically, an introduction by a pretend study doctor to highlight that the system was (1) prescribed by a doctor, (2) the system would be mailed to the patient’s house, and (3) everything they needed to use the system would come in the box. Then, participants were given the opportunity to unbox and familiarize themselves with the product and instructions for use (IFU) as they would at home. Throughout the unboxing and simulated use scenarios, participants also had the opportunity to watch a welcome video and had access to a helpline via a test facility telephone or their phone, with a MedRhythms support representative providing remote assistance.

For simulated use scenarios, participants were observed while they completed realistic task sequences needed for a walking session including system setup, initiating a session, ending a session, and charging the components for future use (Table 2). During this activity, the moderator evaluated performance twice—once with the headset available and once without the headset to evaluate the usability and safety of listening via both headset and device speakers. Across the 2 conditions, participants were scored on 4 unique critical tasks with the device, resulting in 8 total critical tasks that were scored. All participants completed the 2 conditions in the same order—first with the headset available and again without the headset. The moderator also noted any observations related to usability and safety on participants’ interaction with the system.

**Table 2:** Description of critical tasks for the simulated use assessment.

Simulated use task	Success criteria
Users clip sensors onto shoes	User puts on sensors from a stable position (such as sitting down)
Adjust the volume so that the volume is loud enough without being uncomfortable	The audio volume is loud enough for the user to hear without being uncomfortable
Stow device—user keeps touchscreen device where it does not impact their environmental awareness and personal stability	The user keeps a touchscreen device where it does not impact their environmental awareness and personal stability
Remove sensors from shoes	Foot sensors are removed from shoes from a stable position (such as sitting down)

As a descriptive example of how a simulated use critical task relates to safety, the “adjusting volume” task in the uFMEA is tied to these hazardous situations and potential harms if a user encountered a use error when (1) the user cannot hear safety cues in their environment, which may lead to the potential harm of kinetic impact, (2) the user is exposed to an acoustic energy condition that could result in hearing discomfort, or (3) the user is unable to hear instructions and therapy which could result in a delay of therapy.

After the simulated use scenario, participants were asked to use the instructional materials provided (eg, IFU and welcome video) to answer 4 questions to evaluate knowledge of critical information (Table 3). This set of critical tasks assessed the participant’s ability to recall critical information from the materials.

**Table 3:** Description of the critical tasks for the knowledge assessment. Participants were asked questions that assessed their knowledge of critical information.

Question	Success criteria
According to the materials, what environments are appropriate for using StridePlus in?	Participant mentions: Home; Track; YMCA; or flat outside space such as a sidewalk or park Flat and level surfaces Locations where you can walk at least 20 steps in a straight line before turning Not for use on treadmills
According to the materials, what environments are NOT appropriate for using StridePlus in?	Participant mentions: Treadmill Places with icy, steep, wet, or dark terrain Places with obstacles in your path like furniture, many people, traffic, and so on Loud places that will compete with the device audio or interfere with situational awareness
According to the materials, what is the proper way to put on and take off the foot sensors?	Participant mentions: Sitting down, being in a stable position, or asking someone else to put them on
According to the materials, what footwear should you use with this system?	Participant mentions: Well-fitted, supportive, OR recommended by a physician Additional acceptable answers include lace-up or Velcro-strap style sneakers. Avoiding wearing flip-flops, slippers, clogs, and any slip-on style shoe

Finally, 7 comprehension tasks were used to evaluate whether participants fully understood key safety information (Table 4) by observing safety icons and demonstrating an understanding of safety statements.

**Table 4:** Description of critical tasks for comprehension assessment. The participant was asked to demonstrate their understanding of the safety icon and statements.

Statement	Success criteria
DO NOT use [InTandem] if any of the system materials are damaged (to prevent risk of electric shock)	Demonstrates understanding to not use damaged materials
To prevent the risk of injury from a slip, trip, or collision: attach and remove the foot sensors from your shoes from a stable position such as sitting down	Demonstrates understanding to put sensors on from a stable position
DO NOT use [InTandem] with the volume very loud or in loud surroundings because it may hide safety cues, such as emergency sirens or cars honking.	DO NOT use [InTandem] with the volume very loud or in loud surroundings because it may hide safety cues, such as emergency sirens or cars honking
Use [InTandem] in a safe walking area. Do not use in poorly lit areas (to avoid potential obstacles) Do not use in areas with obstacles or potential hazards in your walking path, such as tables, chairs, rugs, pets, pedestrians, or traffic Do not use in areas with uneven, wet, or icy terrain	Demonstrates understanding to not perform walking sessions on uneven surfaces (stairs, ramps, or wet surfaces) or in crowded locations with many obstacles
To help [InTandem] work properly and ensure accurate step detection from the foot sensors: Use [InTandem] in areas where you can walk at least 20 steps in a straight line before turning. Avoid locations with high noise levels that may limit your ability to hear system instructions and music. Avoid uneven walking surfaces (such as frequent stairs, ramps, or hiking trails) Do not use [InTandem] on a treadmill	Demonstrates understanding to perform walking sessions in a flat environment, not on a treadmill
Do not walk while looking at the [InTandem] touchscreen device	Demonstrates understanding to not view their device during walking sessions
Do not continue using [InTandem] if the walking exercise becomes too difficult. Take breaks as needed. A session can be paused and resumed on the touchscreen device screen at any time throughout the session. If you ever become uncomfortable or feel unsafe with the speed of the music, walk at your natural pace. The speed of the music will adjust back to the starting pace of your walking If you experience muscle or joint pain or discomfort that you normally do not have, stop using [InTandem] and consult a medical professional If you experience any abnormal symptoms such as nausea, shortness of breath, or excessive fatigue, stop using [InTandem] and consult a medical professional	Demonstrates understanding to not walk if they become too tired or experience a health issue

Performance of user tasks was scored as “success,” “success with close call,” “success with difficulty,” “use error,” or “not applicable” (definitions provided in Multimedia Appendix 2). Descriptions of use error severity in the uFMEA risk management plan can be found in Multimedia Appendix 3.

### Data Management, Collection, and Analysis

Bold Insight carried out the data collection, management, and analysis. Consent for data collection, use, and sharing of data with the study sponsor (MedRhythms), other authorized persons, and regulatory agencies was collected. Data were deidentified and each participant was given a unique participant code for data collection and analysis purposes. Data included demographic and background information (eg, age and gender), personal health information (eg, medical history) and study visit data, such as (1) participant task performance data; (2) instances of test administrator assistance; (3) system device malfunctions; (4) reported root causes; (5) interview responses; (6) completion of noncritical tasks; and (7) use of the instruction manual, setup video, and helpline during handling scenarios. Sessions were video and audio recorded, and the results were documented and analyzed in a test report formatted to conform to FDA guidance [24].

Use errors were analyzed in two ways: (1) as a percent of participants who successfully completed the critical tasks without use errors for each of the 3 evaluation methods and (2) as the error rates observed out of all opportunities for errors for each of the 3 evaluation methods. For any use errors, close calls, and operational difficulties associated with critical tasks, the moderator probed for underlying root causes of the observed use-related event. Root cause analysis consisted of asking in-depth and open-ended questions to understand the source of any participant confusion or use errors. Documented use–related events were discussed with a cross-functional team to determine if additional design improvements were necessary to mitigate risks. Difficulties and close calls were calculated as the frequency with which they occurred for each task type.

## Results

Each participant completed 8 simulated use tasks, 4 knowledge assessments, and 7 comprehension assessments. In total, 93% (14/15) of participants successfully completed the simulated use tasks associated with at-home use of InTandem. Out of the 120 opportunities for error in the simulated use (15 participants × 8 simulated use tasks), there was 1 error observed, resulting in an error rate of 0.8% (1/120). The 1 use error was observed during the system setup involving the placement of sensors onto shoes. This participant (P01) initially expected that the charging cables should remain attached to the sensors on one’s shoes while using the product and attempted to keep the charging cables attached at the beginning of the walking task. The moderator paused the participants to prevent them from walking with the charging hub. Root cause analysis revealed that due to a software bug, the participant was able to hear the welcome video but could not view the video, which describes and demonstrates the proper way to attach the sensors for a walking session. However, this mitigation strategy of watching and listening to the video was not fully available. During the root cause investigation, the participant indicated that the video would have been helpful to see during setup. Therefore, the root cause for this use error was ascribed to the software bug, which was subsequently corrected. The participant also did not review information in the IFU that showed how the sensors should be attached to the shoes. The severity of the associated use error was categorized as moderate, with the possibility of a slip, trip, or fall resulting in harm. Aside from this, all participants were successful in completing the walking task, and no instability or loss of path was observed. During the course of the simulated use evaluation, no new critical tasks were identified, and no issues were experienced with the noncritical tasks.

During the knowledge assessments, 93% (14/15) of participants were able to find and correctly interpret information in the instructional materials, including the IFU and welcome video, to answer the questions. Out of the 60 opportunities for error in the knowledge assessments (15 participants × 4 knowledge assessments), there was 1 error observed, resulting in an error rate of 2% (1/60). One participant (P013) experienced a use error in which they were unable to specify where the information about appropriate environments could be found in the IFU. The participant assumed that guidance on appropriate environments would be placed toward the end of the IFU after the sections that outlined how to set up the system and did not look for information at the beginning of the IFU where it was located. However, this participant was able to correctly state the appropriate use environments for the system; therefore, after root cause analysis was conducted, the research team determined that additional mitigation strategies were not required for this use error.

Similar to their performance on simulated use tasks and knowledge assessments, participants showed high success with the comprehension assessments. In total, 93% (14/15) of participants understood warning statements associated with critical tasks presented in the IFU. Out of the 105 opportunities for error in the comprehension assessments (15 participants × 7 comprehension assessments), 1 error was observed, resulting in an error rate of 1% (1/105). The 1 use error was observed related to the warning: “DO NOT use [InTandem] with the volume very loud or in loud surroundings because it may hide safety cues, such as emergency sirens or cars honking.” The participant (P10) believed that the statement was instructing them to use InTandem in quiet places so that outside noises would not interfere with the instructions. However, the task’s success criteria required participants to specifically mention the ability to hear safety cues in their environment. Comprehension of this information is important because people need to maintain awareness of their surroundings. If the volume is too high, there could be a risk of not hearing an environmental safety cue such as a potential collision that may be outside of their view. Ultimately, the participant demonstrated their complete understanding of the statement, so no additional mitigations were required to further reduce the risk of a slip, trip fall, or kinetic impact.

With respect to “close calls” and “difficulties,” 0 “close calls” and 1 “difficulty” in the knowledge assessment were observed. The 1 “difficulty” was due to a participant (P01) who had trouble understanding the knowledge task question “According to the materials, what is the proper way to put on and take off the foot sensors?” However, the research team categorized this as a “success with difficulty” because during root cause probing, it was evident the participant understood the proper way to put on and take off the foot sensors.

## Discussion

### Principal Findings

The goal of this study was to validate that participants representative of InTandem’s intended use population can safely and effectively use InTandem, through the completion of critical tasks, and demonstration of knowledge and comprehension of materials. Overall, the occurrence of use errors was observed to be extremely low. For the 3 use errors that occurred in the simulated use tasks, knowledge assessments, and comprehension tasks, root causes were attributed to a software bug and expectations about product use and instructional materials. For the software bug–related use error, we cannot be certain that the use error would not have happened even if they had seen the video while the components were attached to the charging cables. However, the order of events as well as the record of the participant having suggested the video would have helped suggest to the research staff that the software bug was the root cause. The software bug was subsequently corrected. Regarding expectations about product use and instructional materials, although 1 participant expected information about appropriate walking environments to be in a different part of the IFU, no additional mitigations were deemed necessary because they were able to accurately state the appropriate environments for use, and this feedback was not prevalent across the study. In addition, for the participant who did not specifically mention the ability to hear safety cues in their environment during the comprehension evaluation, they were ultimately able to demonstrate their understanding of the safety statement, and existing risk mitigations were deemed sufficient. With respect to difficulties and close calls, there was only 1 difficulty observed in the knowledge assessment. This participant later exemplified that they knew the correct answer during the root cause investigation. The 3 use errors observed for each of the 3 evaluation methods were experienced by 3 different participants, suggesting that all participants showed a high level of performance in critical tasks associated with the safe and effective use of InTandem.

### Contextualization With InTandem Formative Research

Leading up to this study, over 2 years and 8000 hours of iterative design and testing were conducted (formative research) that tailored the system for use in the home environment by the intended patient population. The formative research was conducted with participants representative of the intended population to ensure that the patient’s voice, needs, and desired experience are represented in InTandem. The formative research also ensured that all tasks associated with potential use errors could be identified and tested, and through design iterations, use errors were reduced to a risk as low as reasonably possible, culminating in a robust product design. The methods of this study align with best practice human factors engineering and user-centered design, which are critical to the development of impactful evidence-based rehabilitative systems that meet an unmet medical need.

### Strengths and Limitations

This study contains important strengths in the study methodology. For example, this research was conducted after extensive formative testing that used a patient-centric and iterative design approach with multiple rounds of design refinement, which helped address the needs of the device users. Second, a third party conducted the study to help mitigate potential bias. Related to the device itself, a high percentage of individuals were successful in completing critical tasks, even though the majority of participants enrolled indicated that they were not “very comfortable” using technology, which suggests that InTandem is intuitive to use. These strengths add to the compounding evidence for InTandem to be used by the intended population in the intended environment for use. The accumulated evidence for InTandem includes a feasibility study that resulted in clinically relevant improvements in speed over 1 and multiple sessions [30] and a reduction in the energetic cost of walking along with improved gait asymmetries [31]; a longitudinal RCT of safety and clinical efficacy of InTandem [32]; and a budget impact model that estimates cost savings to payers [33]. Furthermore, MR-001 (InTandem) was designated as a breakthrough device by the FDA in 2020 [34], which underscores both the unmet need to address persistent walking deficits of people who are in the chronic stroke phase of recovery in an accessible and effective manner and the merit and opportunity that InTandem offers them. With the findings presented in this paper and the holistic evidence base of InTandem to improve walking impairment in the chronic stroke population, there is a convincing rationale for its place as a rehabilitation option available to patients.

This study is not without limitations. For example, this study included a walking component that only lasted for 5 minutes as opposed to the 30-minute sessions that would occur in real-world use. This was intentional since the aims of the study were not to assess the treatment effect of the intervention which was assessed in both the feasibility study and the RCT but rather to assess the usability of the system by the intended population in the intended environment. In addition, this research focused on only critical tasks involved in the use of InTandem, and although noncritical tasks were necessarily experienced as part of the testing protocol, they were not scored. Another limitation is that gait impairment was not included as a screening criterion, which would align the study population even closer to the intended population. However, even without a screening criterion, 87% (13/15) of participants enrolled experienced walking impairment. Given the fact that the focus of this study was on using the product and not on the efficacy of the intervention to improve walking, the need for representative walking impairment may be less critical than if the focus was on walking outcomes. Another limitation of the study is that although all participants were recruited and screened for not needing a caregiver to perform the activities, during the warm-up questions, 1 participant indicated that their caregiver would help with some tasks and was subsequently allowed to have their caregiver join them in the room. InTandem was designed to be used independently without a caregiver present, and none of the critical task performances were affected by the inclusion of the caregiver, suggesting InTandem can be used safely and effectively as designed. Another limitation is that there were no participants who had less than a high school education enrolled in this study, which would have reflected a potentially lower literacy population. Finally, racial and ethnic diversity was not an explicit recruitment criterion, and such data were not captured in this study. Future work on InTandem will benefit from newly implemented product research standards to increase racial diversity in studies.

### Conclusions

This paper describes the methods and results of the patient-centered process undertaken to validate the safety and effectiveness of the use of InTandem at home by people living with chronic stroke. In this validation study, people in the chronic phase of stroke recovery were able to safely and effectively use InTandem in the intended setting. This validation study contributes to the overall understanding of residual use–related risks, in consideration of the established benefits of InTandem.

## Appendix

Multimedia Appendix 1 Definitions and examples of common terminology used.

Multimedia Appendix 2 Definition of performance scores.

Multimedia Appendix 3 Severity of potential harm definitions.

## Abbreviations

FDA: Food and Drug Administration
IFU: instructions for use
RAS: rhythmic auditory stimulation
RCT: randomized controlled trial
uFMEA: use failure mode and effects analysis
