# Correction: Validating the Safe and Effective Use of a Neurorehabilitation System (InTandem) to Improve Walking in the Chronic Stroke Population: Usability Study

**DOI:** 10.2196/56041

**Published:** 2024-02-21

**Authors:** Kirsten Elisabeth Smayda, Sarah Hodsdon Cooper, Katie Leyden, Jackie Ulaszek, Nicole Ferko, Annamaria Dobrin

**Affiliations:** 1 MedRhythms Portland, ME United States; 2 Bold Insight Downers Grove, IL United States; 3 Eversana Burlington, ON Canada

In “Validating the Safe and Effective Use of a Neurorehabilitation System (InTandem) to Improve Walking in the Chronic Stroke Population: Usability Study” (JMIR Rehabil Assist Technol 2023;10:e50438) the authors made three linguistic improvements, added one missing acknowledgment, and changed the corresponding author:

In the first sentence of the Discussion, the original text reads as:

The goal of this study was to validate that participants representative of InTandem’s intended use population can safely and effectively use InTandem, through the completion of critical tasks, and demonstrate knowledge and comprehension of materials

The word “demonstrate” should be “demonstration of” to align to the phrasing of “completion of critical tasks” and will now read as:

The goal of this study was to validate that participants representative of InTandem’s intended use population can safely and effectively use InTandem, through the completion of critical tasks, and demonstration of knowledge and comprehension of materials.

In the first paragraph of the “Strengths and Limitations” section, the original text reads as:

The accumulated evidence for InTandem includes a feasibility study that resulted in a clinically relevant improvements in speed…

The authors removed the “a” between “in” and “clinically relevant” and the text now reads as:

The accumulated evidence for InTandem includes a feasibility study that resulted in clinically relevant improvements in speed...

In the “Background on Formative Testing” section, the original text reads as:

...(2) the identification of which interactions with the product users needed the most education and were less immediately intuitive out of the box.

The text should include “on” after “education” and will now read as:

…(2) the identification of which interactions with the product users needed the most education on and were less immediately intuitive out of the box.

The authors neglected to acknowledge a colleague in the Acknowledgments section which originally read as:

This work acknowledges the intellectual contributions made by the broader team at EVERSANA and MedRhythms. We thank Chrissy Stack, Jennifer Lavanture, Holly Roberts, Barbara Heikens, and Lauren Steidl for their contributions to and coordination of this paper, and Eric Richardson for study support during both formative and validation research activities. This work was supported by MedRhythms.

And will now read as:

This work acknowledges the intellectual contributions made by the broader team at EVERSANA and MedRhythms. We thank Chrissy Stack, Jennifer Lavanture, Holly Roberts, Barbara Heikens, and Lauren Steidl for their contributions to and coordination of this paper, Ashley Levesque for her manuscript preparation support, and Eric Richardson for study support during both formative and validation research activities. This work was supported by MedRhythms.

The original corresponding author was Sarah Hodsdon Cooper:

Sarah Hodsdon Cooper, BA

MedRhythms

183 Middle Street

Portland, ME, 04101

United States

Phone: 1 207 233 2373

Email: secooper@medrhythms.com

Has been updated to Kirsten Elisabeth Smayda:

Kirsten Elisabeth Smayda, BA, BM, MM, PhD

MedRhythms

183 Middle Street

Portland, ME, 04101

United States

Phone: 1 207 233 2373

Email: ksmayda@medrhythms.com

In addition, we have updated the author metadata to indicate that authors KES and SHC (first two authors) contributed equally.

The correction will appear in the online version of the paper on the JMIR Publications website on February 21, 2024, together with the publication of this correction notice. Because this was made after submission to PubMed, PubMed Central, and other full-text repositories, the corrected article has also been resubmitted to those repositories.

