# Supplementary material for: Validating the Safe and Effective Use of a Neurorehabilitation System (InTandem) to Improve Walking in the Chronic Stroke Population: Usability Study
Source: JMIR Rehabil Assist Technol. 2023 Nov 20;10:e50438. doi: 10.2196/50438 (PMC10696501; doi:10.2196/50438)
Supplement: Multimedia Appendix 1 [file rehab_v10i1e50438_app1.docx]

Multimedia Appendix 1. Definitions and examples of common terminology used.

| **Term** | **Definition** | **Example in Study** |
| --- | --- | --- |
|  |  |  |
| User Task | Task performed by the user during testing | User puts on headset |
| Critical Task | Tasks that could cause serious harm in the event of a use error scenario; tasks with a severity of “moderate” or greater identified in the uFMEA | User attaches sensors onto shoes |
| Non-Critical Task | Tasks that could not cause serious harm in the event of a use error scenario; tasks with a severity less than “moderate” identified in the uFMEA | Users rate session on survey |
| Use Error | Action or lack of action that was different from that expected by the manufacturer and caused a result that was different from the expected result, was not caused solely by device failure, and did/could result in harm | User places sensors from an unstable position |
| Potential Harm of Use Error | Each use error was reviewed to identify potential hazardous situation users would be exposed to during product use | Slip / Trip/ Fall |
| Risk Mitigation | Risk management measure implemented to eliminate or reduce the risk | Software is being revised prior to launch to eliminate the bug that enabled the welcome video to play without visuals |
| Success Criteria | Criteria used to measure successful completion of task in the validation study | Touchscreen device is powered on |
| Severity of Potential Harm | See categorization in Multimedia Appendix 3. | |

### 
