# Supplementary material for: Validating the Safe and Effective Use of a Neurorehabilitation System (InTandem) to Improve Walking in the Chronic Stroke Population: Usability Study
Source: JMIR Rehabil Assist Technol. 2023 Nov 20;10:e50438. doi: 10.2196/50438 (PMC10696501; doi:10.2196/50438)
Supplement: Multimedia Appendix 2 [file rehab_v10i1e50438_app2.docx]

Multimedia Appendix 2. Definition of performance scores.

| **Score** | **Description** |
| --- | --- |
|  |  |
| Success | Participant was able to complete each task using the device without serious harm to themselves or to others and without moderator assistance. |
| Success with Close Call | Instances in which a participant had difficulty or made a use error that could result in harm, but the participant took an action to “recover” and prevented harm from occurring. |
| Success with Difficulty | Instances in which a participant appeared to struggle to perform a use scenario. This struggle might have been indicated by multiple attempts to perform the use scenario, anecdotal comments about the use scenario’s difficulty, requests for assistance with the use scenario, facial expressions suggesting frustration or confusion, and higher than usual use scenario performance times. |
| Use Error | Participant action or lack of action that was different from that expected by the manufacturer and caused a result that was different from the result expected by the user, was not caused solely by device failure, and did or could result in harm. |
| Not Applicable (N/A) | Participant never attempted a task due to time constraints or a previous error that did not allow him/her to attempt a task. |
