# Supplementary material for: Validating the Safe and Effective Use of a Neurorehabilitation System (InTandem) to Improve Walking in the Chronic Stroke Population: Usability Study
Source: JMIR Rehabil Assist Technol. 2023 Nov 20;10:e50438. doi: 10.2196/50438 (PMC10696501; doi:10.2196/50438)
Supplement: Multimedia Appendix 3 [file rehab_v10i1e50438_app3.docx]

Multimedia Appendix 3. Severity of potential harm definitions.

| **Severity Level** | **Description** |
| --- | --- |
| 5 - Catastrophic | Serious injury (irreversible) or death of the patient or user; very severe negative effect on the environment. |
| 4 - Critical | Serious injury (reversible) to the patient or user. For example, this includes the need for a more invasive procedure such as surgery or increases case complication to fully treat the injury. |
| 3 - Moderate | Moderate injury to the patient or user. For example, this includes actions taken to treat the patient or user within the scope and type of treatment already in progress. |
| 2- Minor | Minor injury to the patient or user. For example, this includes prolonging or delays to a clinical procedure that do not pose a risk of greater injury to the patient or user. |
| 1 - Insignificant | No/virtually no injury to patient or user. |
